# Association of intestinal microbiota markers and dietary pattern in Chinese patients with type 2 diabetes: The Henan rural cohort study

**DOI:** 10.3389/fpubh.2022.1046333

**Published:** 2022-11-16

**Authors:** Guanjun Wang, Quanjun Lyu, Tianyu Yang, Songyang Cui, Kailin Niu, Ruohua Gu, Yan Li, Jia Li, Wenguo Xing, Linlin Li

**Affiliations:** ^1^Department of Nutrition and Food Hygiene, School of Public Health, Weifang Medical University, Weifang, China; ^2^Department of Nutrition, The First Affiliated Hospital of Zhengzhou University, Zhengzhou, Henan, China; ^3^Department of Epidemiology and Health Statistics, College of Public Health, Zhengzhou University, Zhengzhou, Henan, China

**Keywords:** T2DM, gut microbiota, 16S rDNA, diet, dietary pattern

## Abstract

Studies on intestinal microbiota in Chinese type 2 diabetes mellitus (T2DM) patients are scarce and correlation studies with dietary intake are lacking. The case-control study included 150 participants (74 T2DM patients and 76 controls) and microbiome analysis was performed using 16S rDNA sequencing. Principal component analysis was used to determine dietary patterns and correlation analysis was used to evaluate the associations between microbiota diversity, T2DM indicators and dietary variables. Compared to controls, the T2DM group had different gut flora characteristics, including lower alpha diversity, higher Firmicutes/Bacteroidetes ratios, statistically significant beta diversity and other specific bacterial species differences. Gut microbiota was associated with several diabetes-related metabolic markers including HOMA2-β, fasting plasma glucose, HbA1c and fasting insulin. Significant associations were also observed between dietary intake pattern and gut flora. The animal foods pattern scores were positively correlated with the relative abundance of the phylum Fusobacteria, and the vegetarian diet pattern scores were positively correlated with the relative abundance of the phylum Actinobacteria. Phylum Actinobacteria mediated the association of vegetarian diet pattern with fasting insulin and HOMA2-β (all *P* < 0.05). Composition of intestinal microbiota in Chinese T2DM patients differs from that of control population, and the intestinal flora is affected by dietary intake while being associated with several diabetes-related metabolic markers. The gut microbiota may play an important role in linking dietary intake and the etiology of T2DM.

## Introduction

For Almost 500 million people worldwide have diabetes, and it is expected to exceed 600 million by 2030 and 800 million by 2045 with the majority of them being T2DM, according to International Diabetes Federation (1). T2DM is a complex chronic metabolic disease, and its occurrence is associated with genetic and various environmental factors. Previous studies have shown that intake of specific types of food was associated with the risk of T2DM (2, 3). Recent studies also suggest that the development of T2DM may also be associated with altered intestinal microbiota and that dietary intake might play a role in the process (4).

Several previous studies have attempted to explore the relationship between intestinal microbiota, dietary intake and T2DM. A number of experimental animal studies have shown that consumption of specific dietary components can alter the structure and function of the murine gut microbial community, and gut microbes are associated with the metabolism of substances which play an important role in the regulation of food and energy intake, glucose tolerance, and insulin sensitivity ((5–8)). Similar results have been observed in observational studies of population where intestinal bacteria affect the immune and metabolic activities of the host (9, 10). In addition, gut microbiota are associated with glucose regulation, insulin resistance, and glucose tolerance, and these results have been confirmed by various animal studies and population research from Europe (11–14). These findings suggest that changes in the composition and function of the intestinal microbiota are associated with T2DM and may be involved in the relationship of T2DM with dietary factors.

China has a large number of diabetic and pre-diabetic patients, and diabetes has become a critical health issue (15). Given the special dietary habits of Chinese people and the differences in intestinal microbiota indicators for T2DM between Chinese and European or American populations (11, 16–19), the results of studies in other countries may not be generally applicable to Chinese people. Thus, the study of the relationship between intestinal microbiota, T2DM and dietary factors is of great importance for the prevention and treatment of diabetes in China (15), and few studies have analyzed these three factors comprehensively. In this study, the case-control study method was used to explore the characteristics of intestinal microbiota in Chinese T2DM patients and to investigate the association between gut microbes, T2DM and dietary intake and the mediating effect of intestinal hygiene products. Our findings could provide basis for the further understanding of the pathogenesis and precise prevention of T2DM.

## Materials and methods

### Study participants

Participants in this community-based study were drawn from the Henan Rural Cohort, a large population-based study of chronic non-communicable diseases in five regions of Henan Province, China (20). In the present study, 74 T2DM patients and 76 matched controls (new-onset T2DM patients randomly selected from 1 site of the cohort; controls matched according to age, gender and other social-demographic features) were included. Of note, unlike other epidemiological or clinical studies, there is no precise formula for calculating the sample size of a survey for intestinal flora studies. Based on the literature of relevant similar studies and previous experience with 16S rDNA gene sequencing analysis, the minimum sample size for each group was 15 cases. The present study has an adequate sample size of 150 subjects, with 74 in the case group and 76 in the control group.

Inclusion criteria were as follows: (1) T2DM patients aged 35 to 79 years old; (2) no intention to change diet or physical activity or lose weight during the study; and (3) informed consent to the study by the participants themselves and their families. Exclusion criteria were as follows: (1) treatment with antibiotics or ingestion of other medications affecting intestinal microbiota within 2 months prior to stool sampling; (2) recent history of diarrhea, inflammatory bowel disease, or other conditions that may affect intestinal microbes; (3) missing information on dietary intake questionnaires or other covariates; and (4) normal energy intake.

Ethical approval was obtained from the Ethics Committee of Zhengzhou University ([2015] MEC (S128)). Written informed consent was obtained from all the participants before the study began.

### Data collection and anthropometric measurements

Basic information about each participant was obtained through a questionnaire administered by trained staffers. Face-to-face interviews were conducted to collect information on demographic characteristics (age, gender, income, marital status and education level), lifestyle behaviors (smoking, alcohol consumption, physical activity and eating habits), personal and family history of disease and other aspects.

In this study, dietary intake was assessed according to the Dietary Guidelines for Chinese Residents and the dietary characteristics of residents in Henan Province using a food frequency questionnaire (FFQ) consisting of thirteen major food items (the reliability and validity of the FFQ have been verified) (21). Food items included staple foods, red and white meat, fish, eggs, dairy, fruits, vegetables, beans, nuts, grains, pickles, and animal oils. Of the major food groups, it was emphasized that Chinses diet had a lower consumption of grains (wheat, rice, fine flour and their products) compared with staple food (Maize, oats, Korghum flour and red thistle), thought to be the primary sources of energy. According to the questionnaire, participants were asked to report the frequency (never, day, week, month, and year) and quantity (kg, g) of food consumed in the past year. For each major food item, dietary recall was assessed using visual photo mapping, allowing participants to assess the specific intake of each food compared to standard doses.

Anthropometric measurements were taken on the same morning as the questionnaire. For weight measurement, participants were dressed lightly without shoes and their weight was rounded to the nearest 0.1 kg; height was measured to the nearest centimeter using a standard ruler. Body mass index (BMI) was then calculated by dividing weight (kg) by height squared (m^2^). After a rest period of at least 5 min, blood pressure was measured three times with an electronic sphygmomanometer (Omron HEM-7071A, Japan) on the right arm at 30-s intervals. The average of the three measurements was used for statistical analysis.

### Blood and fecal samples collection and laboratory analysis

Venous blood samples were taken from overnight fasting participants for laboratory measurements. Fasting plasma glucose (FPG), total cholesterol (TC), triglycerides (TG), high-density lipoprotein cholesterol (HDL-C), and low-density lipoprotein cholesterol (LDL-C) were measured either directly or enzymatically using a ROCHE Cobas C501 automated biochemistry analyzer. Homeostatic model assessments of insulin resistance (HOMA2-IR) and beta-cell function (HOMA2-β) were calculated according to the designed equations based on fasting glucose and insulin levels (https://www.dtu.ox.ac.uk/homacalculator/) (22).

Participants were given a specimen collection kit and stool samples were obtained at their homes. Fecal samples were first stored in their home freezer and then transferred and stored at −80°C until further use. DNA quantity and quality were analyzed using the Nanodrop 2,000 spectrophotometer (Thermo Scientific) and molecular size agarose was estimated by gel electrophoresis.

The V3 and V4 region of the bacterial 16S rRNA gene was amplified from extracted fecal DNA through degenerate primers (338F: 5'-ACTCCTACGGGAGGCAGCA-3', 806R: 5'-GGACTACHVGGGTWTCTAAT-3'). The amplicons were purified and quantified according to manufacturer's protocols. The procedure of polymerase chain reaction (PCR) is as follows: denaturation (2 min at 98°C), followed by 30 cycles consisting of denaturation (30 s at 98°C), annealing (30 s at 50°C), extension (60 s at 72°C) and a final extension at 72°C for 5 min. PCR products were detected by 1.8% agarose gel electrophoresis. The magnetic bead system was used to purify the replicate PCR reactions. Purified PCR amplicons of each sample were mixed, according to the amplicon concentration of samples detected by Nanodrop. The amplicons were sequenced in a single pool in one run with the Hiseq 2,500 platform (250 PE, Illumina).

### Covariate definition

Educational attainment was classified into four categories based on the questionnaire report: illiterate, primary school, junior high school, senior high school and above. Marital status was grouped into two levels, including married/cohabiting and other/widowed. The per capita monthly household income was calculated on a household basis and divided into three categories: < 500 RMB/month, 500–1000 RMB/month and ≥1,000 RMB/month. Smoking and drinking status were both divided into two levels according to whether the subjects have a history of smoking or drinking. Family history of diabetes is defined as any immediate family member (e.g., parent, sibling or child) of the study participant having diabetes. The International Physical Activity Questionnaire (IPAQ) classifies physical activity levels into three levels: heavy physical activity, moderate physical activity and light physical activity.

### Bioinformatics analysis

The raw sequences were processed to concatenate forward and reverse reads using FLASH (version 1.2.11), and resulting sequences were quality filtered by Trimmomatic (version 0.33) and chimeras removed with UCHIME (version 8.1) (23–25). Sequences were aligned through USEARCH (version 10.0) and clustered into OTUs by 97% similarity, and taxonomy was assigned using RDP Classifier (version 2.2) with Silva (Release 132) as a reference base (26–28). The phylogenetic tree was built with PyNAST (version 1.2.2) with the Neighbor-Joining method (29).

Alpha diversity (Chao1 index, Ace index, Shannon index and Simpson index) was calculated using Mothur (version v.1.30). Beta diversity (weighted and unweighted UniFrac distance metrics) were calculated and visualized using R by principal coordinates analysis (PCoA).

Linear discriminant effect size analysis (LEfSe) based on OTU level was performed to find differentially represented features between control and T2DM groups (30).

### Statistical analysis

Given the differences between the two groups compared, normally distributed data were expressed as mean ± standard deviation (SD) and were analyzed using the independent *t*-test, while non-normally distributed data (continuous variables) were expressed as medians and interquartile ranges (IQR) and analyzed using the Wilcoxon rank sum test. The χ2 test was used to test for differences in categorical variables between cases and controls. The alpha diversity was tested with the Wilcoxon rank-sum test among groups. Permutational multivariate ANOVA (PERMANOVA) was performed to test beta diversity dissimilarity. Wilcoxon rank sum test was used to assess the association of relative abundance among each taxonomic level. We used a Spearman correlation analysis to examine the correlations between dietary intake with intestinal microbiota abundance, and dietary intake with diabetes-related metabolic markers. Mediation analysis was conducted using the relative abundance of intestinal microbiota as a mediator of the relationship between dietary intake and diabetes-related metabolic markers to investigate the relevance of significant findings related to type 2 diabetes, with potential covariates adjusted. False discovery rate (FDR) using the Benjamini-Hochberg method was applied to correct for the significant *P*-values. The PCA method was used to extract data and reduce the dimensionality of dietary intake information (in 11 food groups except for pickles and animal oils) and the factors were variationally orthogonal transformed. Identified factors were retained by scree plot, evaluated eigenvalues (>1) and interpretability. The sample adequacy was examined using the Kaiser-Mayer-Olkin (KMO) test for factor analysis (KMO = 0.69 > 0.6, Bartlett's test of sphericity *p* < 0.01). All results were deemed significant if the *p*-value was below 0.05 and data analysis was performed using R software (version 4.0.3; R Project for Statistical Computing, Vienna, Austria).

## Results

### Demographic characteristic

Basic information on participants is shown in Table 1. Among the 150 participants, the mean (SD) age was 59.39 (8.01) years, of which 61.3% were female individuals. The distribution of age, gender, demographic information and socioeconomic background, as well as other lifestyle habits and physical activity data were similar between T2DM and control individuals, for which the differences were not statistically significant. Compared to controls, T2DM participants had higher BMI, WC, FPG, fasting insulin, HbA1c, TC and TG (*P* < 0.05 for all), but no significant differences in HDL-C and LDL-C (Table 1).

**Table 1:** Characteristics of T2DM cases and controls.

**Characteristics**	**T2DM (*n*=74)**	**Controls (*n*=76)**	***P***
Gender (%)			1
Male	29 (0.4)	29 (0.4)	
Female	45 (0.6)	47 (0.6)	
Age (year)	59.46 ± 7.88	59.32 ± 8.18	0.763
Education level			0.831
Illiterate	20 (0.3)	21 (0.3)	
Elementary school	29 (0.4)	26 (0.3)	
Junior high school	20 (0.3)	21 (0.3)	
High school or above	5 (0.1)	8 (0.1)	
Marriage			0.566
Married/cohabiting	64 (0.9)	69 (0.9)	
Other/solitary	10 (0.1)	7 (0.1)	
Income			0.533
< 500 CNY	30 (0.4)	37 (0.5)	
500–1,000 CNY	19 (0.3)	19 (0.3)	
>1,000 CNY	25 (0.3)	20 (0.3)	
Alcohol			0.447
Y	13 (0.2)	9 (0.1)	
N	61 (0.8)	67 (0.9)	
Tobacco			0.888
Y	12 (0.2)	14 (0.2)	
N	62 (0.8)	62 (0.8)	
Family history of diabetes			0.114
Y	5 (0.1)	1 (0)	
N	69 (0.9)	75 (1)	
Physical activity			0.244
Low	16 (0.2)	19 (0.3)	
Moderate	38 (0.5)	45 (0.6)	
High	20 (0.3)	12 (0.2)	
BMI (kg/m^2^)	25.5 ± 3.5	23.5 ± 3.0	< 0.001
WC (cm)	86.8 ± 9.2	80.8 ± 8.9	< 0.001
FPG (mmol/L)	9.3 ± 2.5	5.0 ± 0.4	< 0.001
Fasting insulin (μU/L)	14.5 ± 6.6	11.7 ± 4.3	0.003
HbA1c (mg/dL)	8.3 ± 1.9	5.3 ± 0.3	< 0.001
TC (mmol/L)	5.0 ± 1.0	4.7 ± 0.8	0.036
TG (mmol/L)	2.3 ± 1.6	1.7 ± 0.8	0.002
HDL-C (mmol/L)	1.3 ± 0.3	1.4 ± 0.3	0.331
LDL-C (mmol/L)	3.2 ± 0.93	3.0 ± 0.8	0.180

χ^2^ tests for categorical variables, *t*-test or Wilcoxon rank sum test for continuous variables; Continuous variables are presented as mean±SD, and categorical variables are expressed as *n* (%); CNY, Chinese yuan; BMI, body mass index; WC, waist circumference; FPG, fasting plasma glucose; TC, total cholesterol; TG, Triglyceride; HDL-C, high-density lipoprotein cholesterol; LDL-C, low-density lipoprotein cholesterol.

### Intestinal microbiome profile

A total of 11,529,795 reads were sequenced from 150 samples, and 10,838,351 clean tags were generated after splicing and filtering, averaging 72,256 clean tags per sample. 432 OTUs were obtained from all samples at the 97% similarity level.

**Figure 1:**
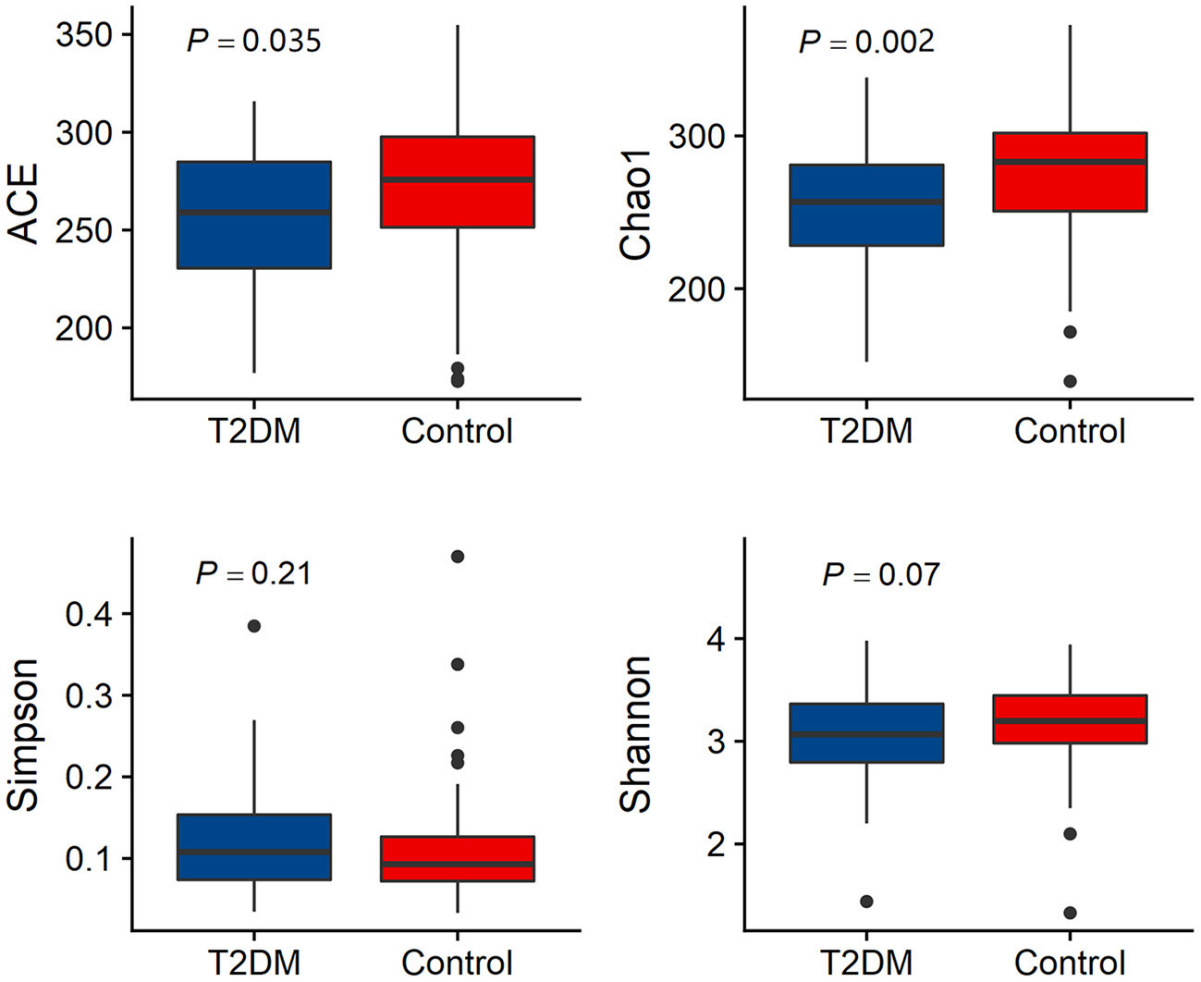
Comparison of alpha diversity (Ace index, Chao1 index, Simpson index and Shannon index) between the gut microbiota of T2DM and controls.

Alpha diversity analysis results are shown in Figure 1. The T2DM group had lower microbial richness compared to the control group participants (ACE index: 259.02 (229.33–284.84) vs. 275.75 (251.29–297.80), *P* = 0.035; Chao index: 256.82 (228.06–282.33) vs. 283.00 (249.28–302.08), *P* = 0.002). There is no difference in microbial diversity indices between T2DM individuals and controls (Simpson index: 0.108 (0.074–0.154) vs. 0.093 (0.072–0.128), *P* = 0.21; Shannon index: 3.068 (2.793–3.364) vs. 3.198 (2.968–3.455), *P* = 0.07) (Figure 1).

**Figure 2:**
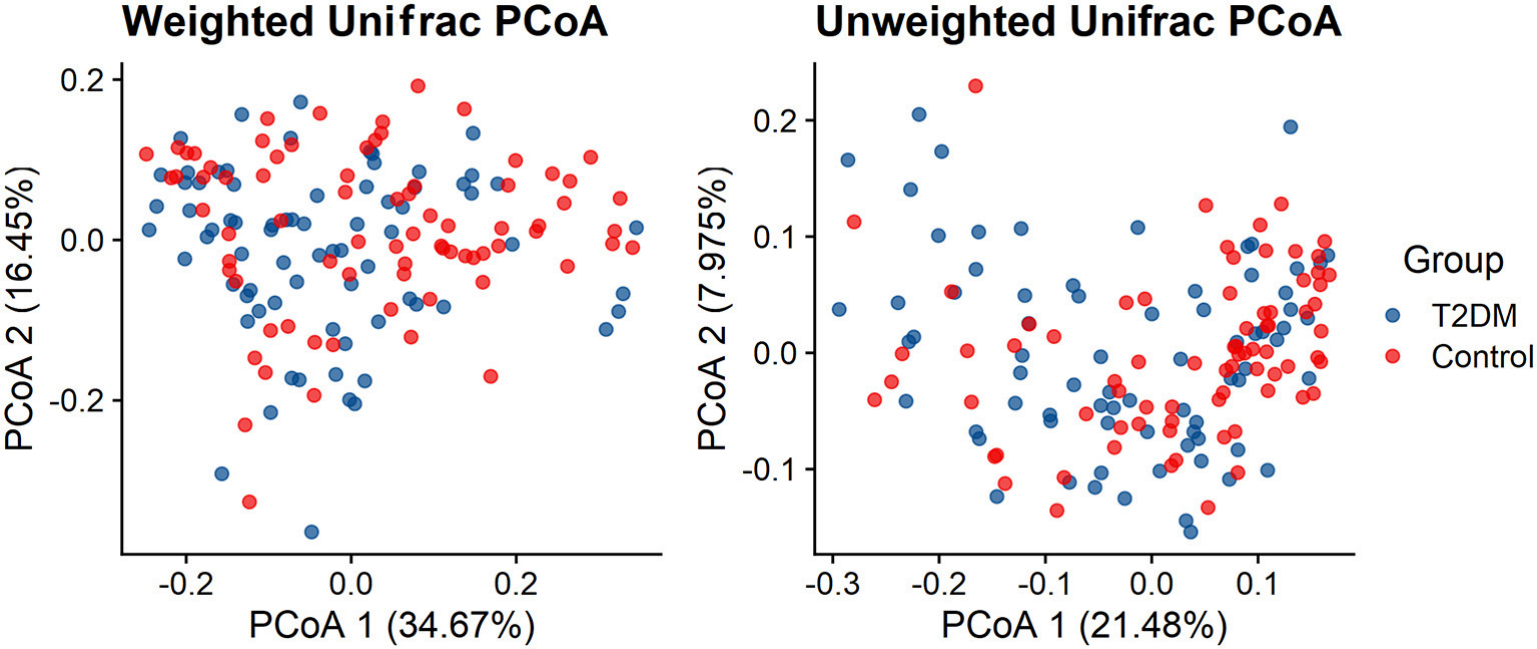
Principal coordinate analysis illustrating the grouping patterns of the T2DM and control individuals based on the unweighted UniFrac and weighted UniFrac distances (both *p* < 0.001).

Both Weighted UniFrac distance metrics and unweighted UniFrac distance metrics to some extent distinguished the intestinal microbiota of T2DM subjects from that of the control subjects. There were significant differences in β-diversity between the T2DM and control groups (*P* < 0.001) (Figure 2).

### Microbial composition difference between T2DM and controls groups

At the phylum level, most of the sequencing reads were classified into four phylum groups (98.61% of total): Firmicutes (65.33%), Bacteroidetes (18.33%), Actinobacteria (8.03%) and Proteobacteria (6.94%). The relative Abundance of each gut microbiota at the phylum level was not significantly different between the T2DM group and the control group. The ratio of Firmicutes/Bacteroidetes was significantly higher in T2DM group [5.69 (2.30–19.14)] than that in the control group [2.89 (1.85–10.62)] (*P* = 0.037). At class level, the Bacteroidia and Melainabacteria showed lower abundances among T2DM cases compared to those of controls, whereas the relative abundances of Erysipelotrichia were higher in T2DM cases than in controls. At the order level, Pasteurellales, Bacteroidales and Gastranaerophilales were less abundant while Erysipelotrichales and Erysipelotrichales were more abundant in the T2DM group compared to non-T2DM controls. At family level, the results showed that the case group had higher abundance of Enterobacteriaceae and Enterococcaceae but lower abundance of Acidaminococcaceae, Clostridiaceae and an uncultured bacterium belonging to Bacteroidales (Supplementary Table 1). At the genus level, 21 genera with significantly different relative abundances were identified between the T2DM and control groups, of which 7 were more abundant in the case group and 14 were more abundant in the control group (All *P* < 0.05) (Table 2).

**Table 2:** Comparison of genus abundance between T2DM cases and controls.

**Genus**	**T2DM**	**Control**	***P***	***P*-adjusted**
Blautia	0.061	0.044	0.001	0.018
Citrobacter	0.002	0.000	0.003	0.032
Clostridium_sensu_stricto_1	0.019	0.026	0.000	0.012
Coprococcus_2	0.005	0.007	0.000	0.013
Enterobacter	0.003	0.000	0.006	0.048
Enterococcus	0.001	0.000	0.006	0.048
Fournierella	0.000	0.000	0.001	0.017
Haemophilus	0.001	0.002	0.008	0.047
Klebsiella	0.027	0.005	0.001	0.015
Lachnospira	0.001	0.002	0.000	0.002
Lachnospiraceae_NK4A136_group	0.005	0.011	0.007	0.048
Paraprevotella	0.000	0.001	0.001	0.017
Phascolarctobacterium	0.006	0.007	0.006	0.044
Roseburia	0.011	0.022	0.001	0.014
Ruminococcaceae_UCG-010	0.001	0.001	0.005	0.048
Ruminococcus_1	0.005	0.007	0.008	0.048
[Eubacterium]_eligens_group	0.009	0.007	0.006	0.046
[Eubacterium]_xylanophilum_group	0.001	0.002	0.006	0.043
Uncultured_bacterium_f_Peptostreptococcaceae	0.000	0.000	0.000	0.015
Uncultured_bacterium_o_Bacteroidales	0.004	0.003	0.001	0.018

**Figure 3:**
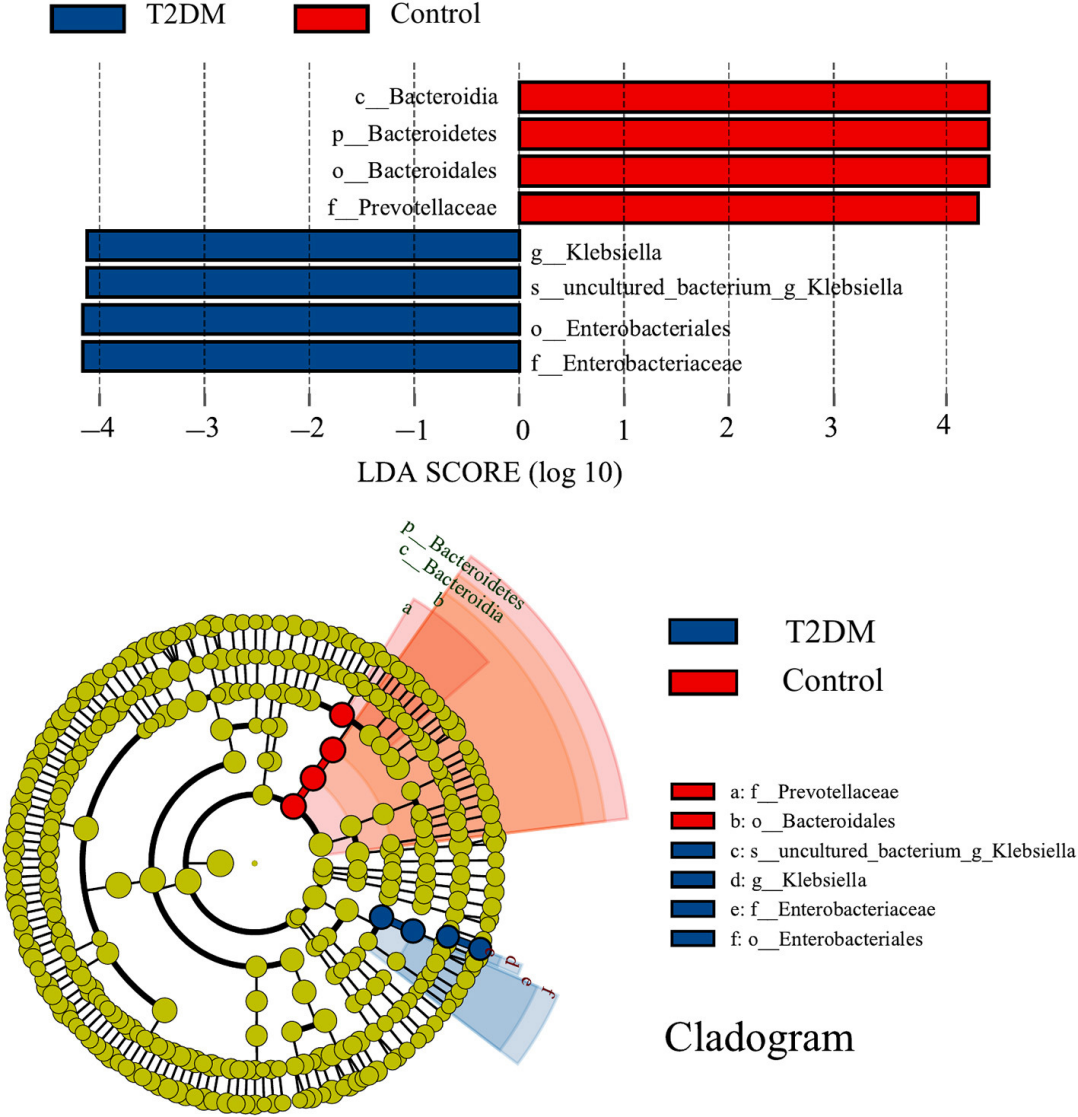
LEfSe analysis and corresponding phylogenetic dendrograms between T2DM and control groups (LDA score ≥ 4). The circles radiating from inside to outside of the phylogenetic dendrograms represent taxonomic levels from phylum to species; each small circle at a different taxonomic level represents a taxon at that level, and the diameter size of the small circles is proportional to the relative abundance size; species with no significant differences are colored uniformly in yellow.

LEfSe analysis identified eight discriminatory features with significant differences in relative abundance between the T2DM and control groups (LDA score ≥4). From the phylogenetic dendrogram, it is clear that the differences mainly occurred in Enterobacteriales and Bacteroidales, which is consistent with the results of the previous analysis of bacterial composition (Figure 3).

### Associations of gut microbiota and T2DM metabolic markers

**Figure 4:**
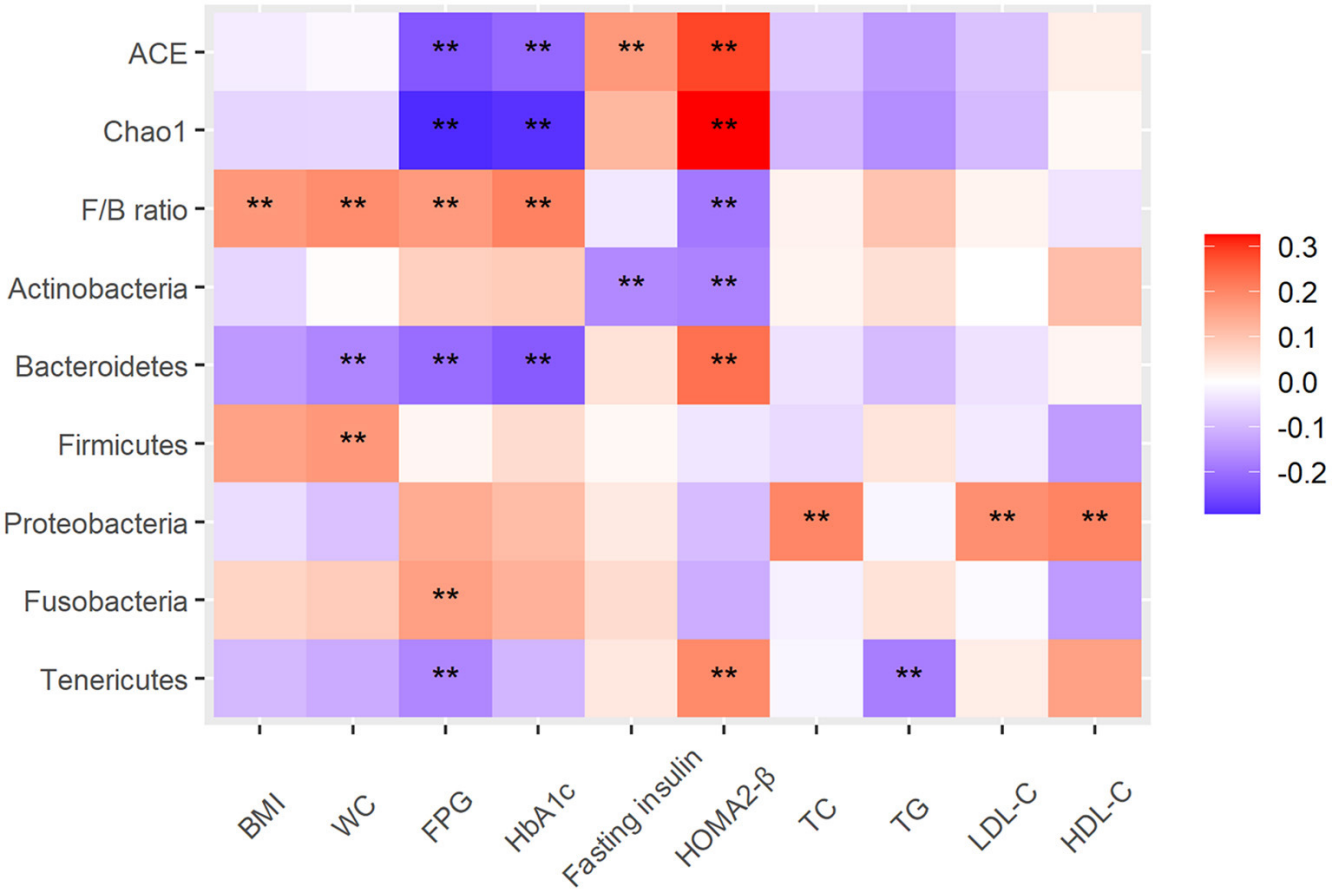
Heat map of the Spearman correlation between intestinal microbiota and type 2 diabetes–related traits. The intensity of the colors represents the degree of association as measured by the Spearman correlation. All significant correlations are marked with an asterisk (*P* < 0.05). Alpha diversity index, ACE, Chao1; F/B ratio, ratio of Firmicutes/Bacteroidetes; BMI, body mass index; WC, FPG, fasting plasma glucose; HOMA2-β, Homeostatic model assessments2 of beta-cell function; TC, total cholesterol; TG, triglycerides; LDL-C, low-density lipoprotein cholesterol; HDL-C, high-density lipoprotein cholesterol.

To explore the relationship between intestinal microbiota, dietary intake and diabetes-related metabolic markers, Spearman's correlation analysis was performed and significant correlations between them were observed. For example, between intestinal microbiota and metabolic markers, alpha diversity (Chao1 index and Ace index) was negatively correlated with FPG and HbA1c, and significantly positively correlated with HOMA2-β; F/B ratio was positively correlated with BMI, FPG and HbA1c, and negatively correlated with HOMA2-β. Phylum Bacteroidetes and Tenericutes were negatively correlated with FPG and significantly positively correlated with HOMA2-β; Fusobacteria was positively correlated with FPG; Proteobacteria was positively correlated with HDL-C, LDL-C and TC (All *P* < 0.05) (Figure 4). And the correlative heat map between metabolic markers and gut microbial genus was displayed in the Supplementary Figure 1.

### Associations of dietary intake and gut microbiota

Correlation analysis of dietary intake and intestinal microbiota revealed that phylum Actinobacteria was positively correlated with dairy intake; Patescibacteria was negatively correlated with beans and grains; WPS-2 was negatively correlated with nuts; and white meat was positively correlated with Firmicutes (Supplementary Figure 2). The correlation analysis of intestinal microbiota at genus level with dietary intake was shown in the Supplementary Figure 3.

Three dietary patterns were obtained by principal component analysis: animal foods pattern, grain pattern and vegetarian diet pattern (patterns were named based on higher food group factor loading) (Supplementary Figure 4). The three factors explained 47% of the variance in the total food intake, with the animal foods pattern explaining 18% and the latter two patterns both accounting for 14% of the variance in food intake. The animal foods pattern was dominated by the intake of white meat, fish, red meat and egg. The grain pattern was characterized by higher intakes of grains (i.e., sweet potatoes/corn), nuts (i.e., peanuts, melon seeds) and legumes, whereas the vegetarian diet pattern was characterized by a predominant intake of staple foods, fruits, dairy products, and vegetables. Correlation analysis of dietary pattern scores with intestinal microbiota indicated that animal foods pattern scores were positively correlated with the relative abundance of the phylum Fusobacteria (*r* = 0.17; *P* = 0.03), and the vegetarian diet pattern scores were positively correlated with the relative abundance of the phylum Actinobacteria (*r* = 0.21; *P* = 0.01).

### The mediating effects of intestinal microbiota

**Figure 5:**
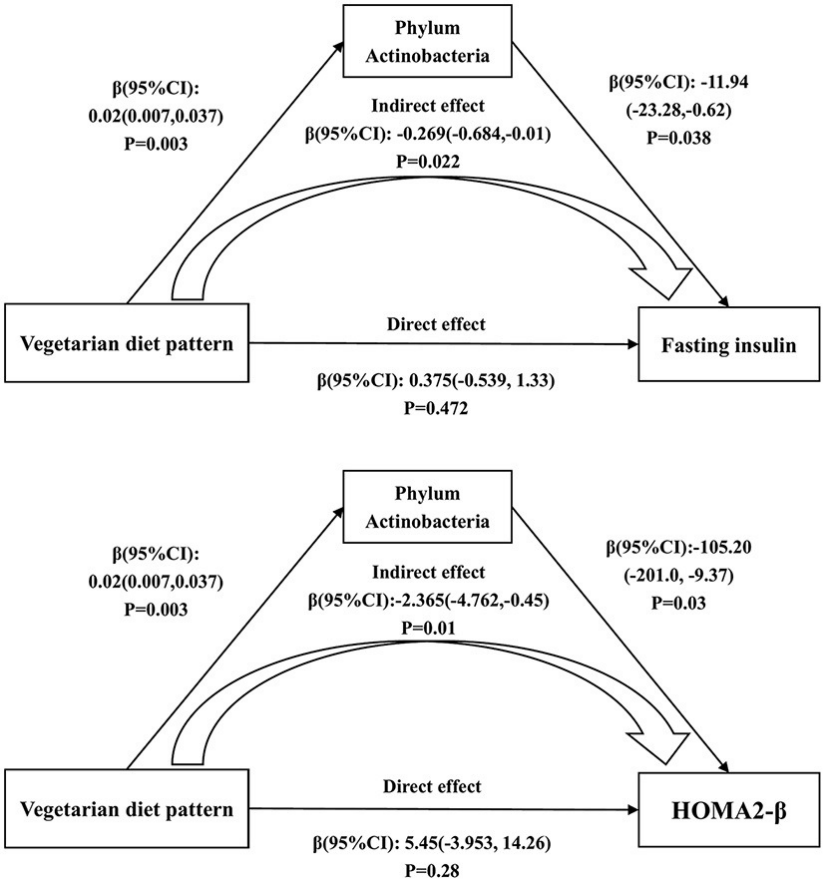
The mediating effects of phylum Actinobacteria on the associations of the vegetarian diet pattern score with fasting insulin and HOMA2-β. Adjusted for age, gender, education level, marriage, income, smoking, alcohol consumption and physical activity.

To explore the relationship among intestinal microbiota, dietary intake and diabetes-related metabolic markers, the mediation analysis was performed and significant association between them were observed. The mediation analysis showed that the association of the vegetarian diet pattern with fasting insulin (Indirect effect; β (95%CI): −0.269 (*-*0.684, −0.01); *P* = 0.022) and HOMA2-β (Indirect effect; β (95%CI): −2.365 (−4.762, −0.45); *P* = 0.01) was mediated by the phylum Actinobacteria (Figure 5).

## Discussion

In this case-control study, we analyzed the intestinal microbiota characteristics of type 2 diabetic patients in a Chinese population, and explored the relationship between intestinal microbiota, type 2 diabetes and dietary intake pattern. To our knowledge, this is the first study to focus on the association between gut microbes, dietary component intake and T2DM in Chinese patients. We found that intestinal microbiota correlated with multiple diabetes-related metabolic markers. ACE index, Chao1 index, and phylum Bacteroidetes were negatively correlated with FPG and positively correlated with HOMA2-β, whereas the F/B ratio was the opposite. In addition, we also had several novel findings that dietary intake also had an impact on gut microbiota, for example, vegetarian dietary patterns were positively correlated with the phylum Actinobacteria and Actinobacteria also mediated the relationship of vegetarian dietary patterns with fasting insulin and HOMA2-β. These findings could help to explain the pathogenesis of T2DM and provide a basis for subsequent intervention, treatment and further research.

Although with different ethnicities, geographic locations, and habitual diets, our findings of intestinal microbiota characterization in Chinese T2DM patients agree well with findings from other populations (31, 32). T2DM patients had lower gut microbiota ACE index and Chao index than the control population; both the ACE index and the Chao index were composite indicators of richness and evenness. Correlation analysis also showed that alpha diversity was negatively correlated with FPG and HbA1c and positively correlated with HOMA2-β. We note that the F/B ratio was higher in diabetic patients and it was positively correlated with BMI, WC, FPG and HbA1c, which is similar to results of an animal experiment and a Ukrainian population study (33–35), suggesting that the F/B ratio is associated with obesity and glucose metabolism, and can be used as a marker for intestinal abnormalities in obese patients. In terms of specific microbial categories, we found that the relative abundance of the phylum Proteobacteria was positively correlated with blood lipid composition, whereas Tenericutes was negatively correlated with FPG and TG and positively correlated with islet secretory function. These findings are further supported by other observational and interventional studies from Chinese and European populations, revealing adverse impacts of Proteobacteria and the probiotic effects of Tenericutes (36–39). All these findings suggest that intestinal microbiota can be used as an entry point for further exploration of T2DM pathogenesis and even diagnosis and treatment.

Other significant findings of our study include the diet's impact on the regulation, structure and diversity of the intestinal microbiota. Diet is an important factor in the regulation of intestinal microbiota and has an important impact on the structure and diversity of the microbiota (4). Several studies have shown that dietary intake plays an important role in the formation of intestinal microbiota and the maintenance of intestinal health (4, 9, 10, 40); for example, dietary fiber, vegetarian dietary patterns, and higher dietary quality have promotive effects on beneficial intestinal bacteria, while high-fat or high-energy diets have detrimental effects (7, 37, 41–44). In this study, we discovered that the dietary pattern of animal foods is positively correlated with the relative abundance of Fusobacteria. Fusobacteria is a common oral microbiota, and studies have shown that Fusobacteria found in the gut is associated with cancer, colitis and T2DM, possibly related to its influence on inflammation and immune responses (45–49). Another study also found similar relationships between animal food intake and Fusobacterium (47). In addition, it was also found that Fusobacteria was positively correlated with beans intake, and negatively correlated with fat intake, seems to be in conflict with the opinion above (50). That means how Fusobacterium can be regulated by dietary factors, even as an intervention or treatment method, is worthy of further study.

Another notable finding from our study is the effects of a vegetarian dietary pattern. Results suggest that the vegetarian diet pattern was positively correlated with Actinobacteria, while Actinobacteria was negatively correlated with FPG and HOMA2-β. Actinobacteria mediated the relationship of vegetarian diet pattern with fasting insulin and HOMA2-β. The vegetarian diet pattern includes ingredients such as vegetables and fruits rich in dietary fiber, which contribute to the growth of fiber-fermenting bacteria. Actinobacteria is the dominant intestinal micromicrobiota in most mammals, and those detected in the gut are usually beneficial, such as Streptomyces and Bifidobacterium, which can ferment fiber and secrete bioactive metabolites including butyrate, lactic acid and B vitamins (51, 52). Based on these properties, a number of Actinobacteria strains are considered to be a probiotic and are added to yogurt or other fermented foods to promote human health (53). Although no studies have reported this mediating role of intestinal microbiota modulating the relationship between vegetarian diet and insulin metabolism indicators, similar direct association results between vegetarian diet with intestinal microbiota and intestinal microbiota with insulin metabolism indicators have been reported in other population studies and interventional trials or animal experiments (36, 40, 41, 48). However, our study found only superficial associations, and further and more robust studies are needed to investigate deeper mechanisms.

Our study has several strengths. First, our study is the first observational study to explore comprehensively the association between dietary intake, intestinal microbiota and T2DM risk. Second, T2DM patients included in this study were all new-onset diabetes patients so as to avoid recall bias and changes in eating habits due to illness. Our study also has its potential limitations that should be taken into account when interpreting the results. This study is a case-control study that cannot yield an accurate causality, so caution is needed in the interpretation of the causality relationship. Second, this study only explored the effects of dietary factors and intestinal microbiota on T2DM patients, while other factors such as lifestyle and socioeconomic conditions were not addressed. Third, the dietary intake data were obtained by the respondents' review and assessment of their food intake in the past year, with some recall bias. Nevertheless, the reliability and validity of this food frequency questionnaire were also assessed by our team. Finally, this study used 16S rDNA sequencing of the intestinal flora, whereas the latest macro-genome sequencing technology can provide more accurate and richer analysis, and the combination with analysis of metabolic substances and functional pathways of the intestinal flora can provide more in-depth and convincing results. Thus, subsequent large metagenome cohort studies, with full consideration of lifestyle, dietary habits, socioeconomic and other factors that may influence gut microbiota are needed to elucidate the relationship between intestinal microbiota, dietary intake and T2DM.

## Conclusion

In summary, in Chinese patients with T2DM, the intestinal microbiota was significantly different from normal individuals, with lower alpha diversity, higher F/B ratio and significantly different beta diversity. Several metabolic markers associated with diabetes, such as HOMA2-β, FPG, HbA1c and fasting insulin, were significantly associated with intestinal microbiota. In addition, there were significant correlations between dietary intake and intestinal microbiota. Intestinal microbiota can, to some extent, explain the relationship between diet and diabetes. These findings could expand our insights into the mechanisms of diet-induced diabetes.

## Data availability statement

The raw data supporting the conclusions of this article will be made available by the authors, without undue reservation.

## Ethics statement

The studies involving human participants were reviewed and approved by Zhengzhou University ethics committee. The patients/participants provided their written informed consent to participate in this study.

## Author contributions

GW: writing—original draft preparation. QL, SC, and TY: data analysis and validation. TY and SC: writing—review and editing. KN and RG: investigation. YL, JL, WX, and GW: conceptualization. LL: project administration and supervision. All authors have read and agreed to the published version of the manuscript.

## Funding

This research was funded by the Key R&D and promotion projects in Henan Province (Grant no. 212102310195) and the Natural Science Foundation of Henan Province (No. 182300410010). The funding sources had no involvement in study design, collection, analysis, interpretation of data, writing of the report and the decision to submit the article for publication.

## Conflict of interest

The authors declare that the research was conducted in the absence of any commercial or financial relationships that could be construed as a potential conflict of interest.

## Publisher's note

All claims expressed in this article are solely those of the authors and do not necessarily represent those of their affiliated organizations, or those of the publisher, the editors and the reviewers. Any product that may be evaluated in this article, or claim that may be made by its manufacturer, is not guaranteed or endorsed by the publisher.
