# Supplementary material for: Association of intestinal microbiota markers and dietary pattern in Chinese patients with type 2 diabetes: The Henan rural cohort study
Source: Front Public Health. 2022 Nov 16;10:1046333. doi: 10.3389/fpubh.2022.1046333 (PMC9709334; doi:10.3389/fpubh.2022.1046333)
Supplement: Supplementary file 1 [file Data_Sheet_1.docx]

Supplementary Material

# Supplementary Figures and Tables

## Supplementary Figures

##
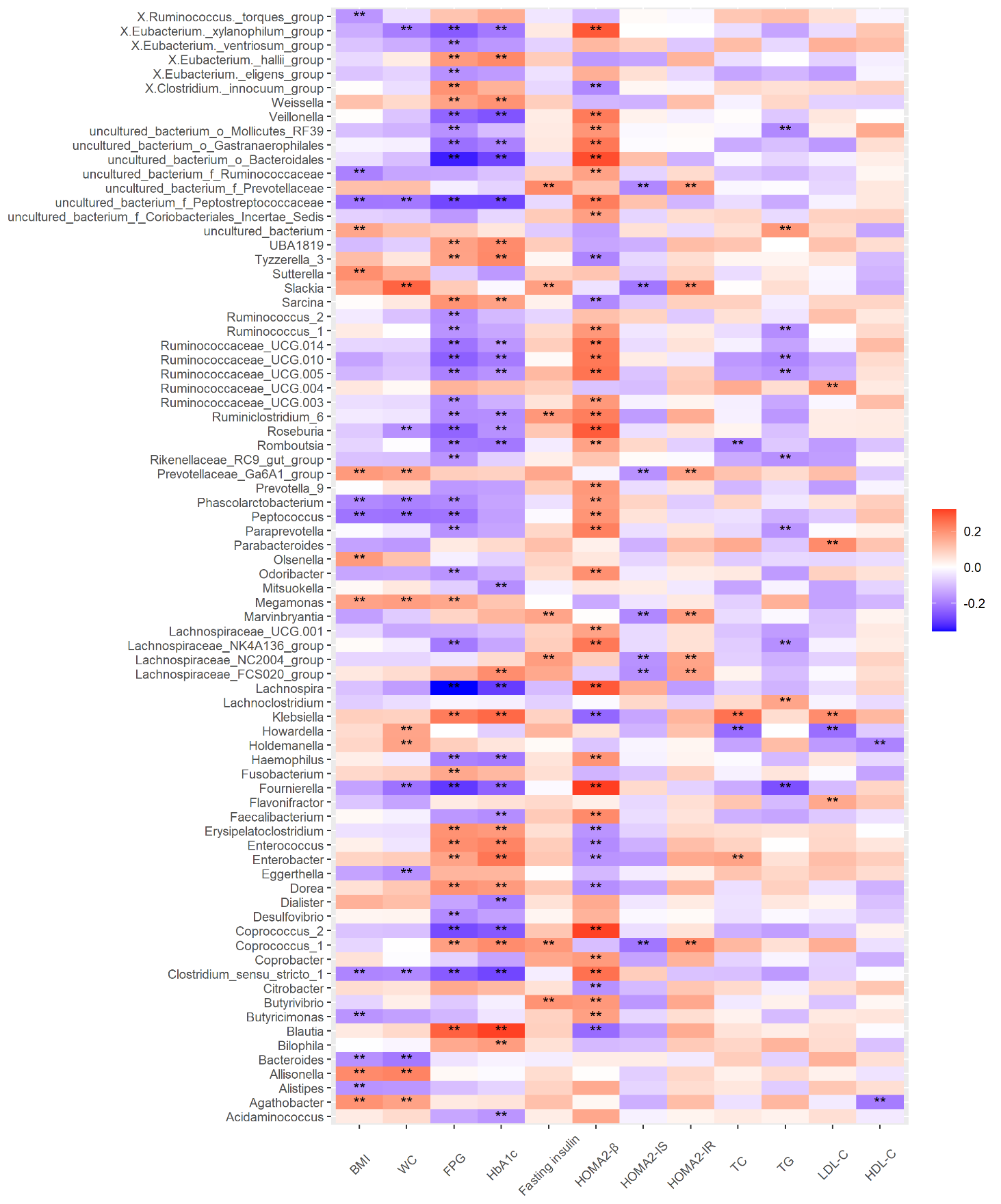


##
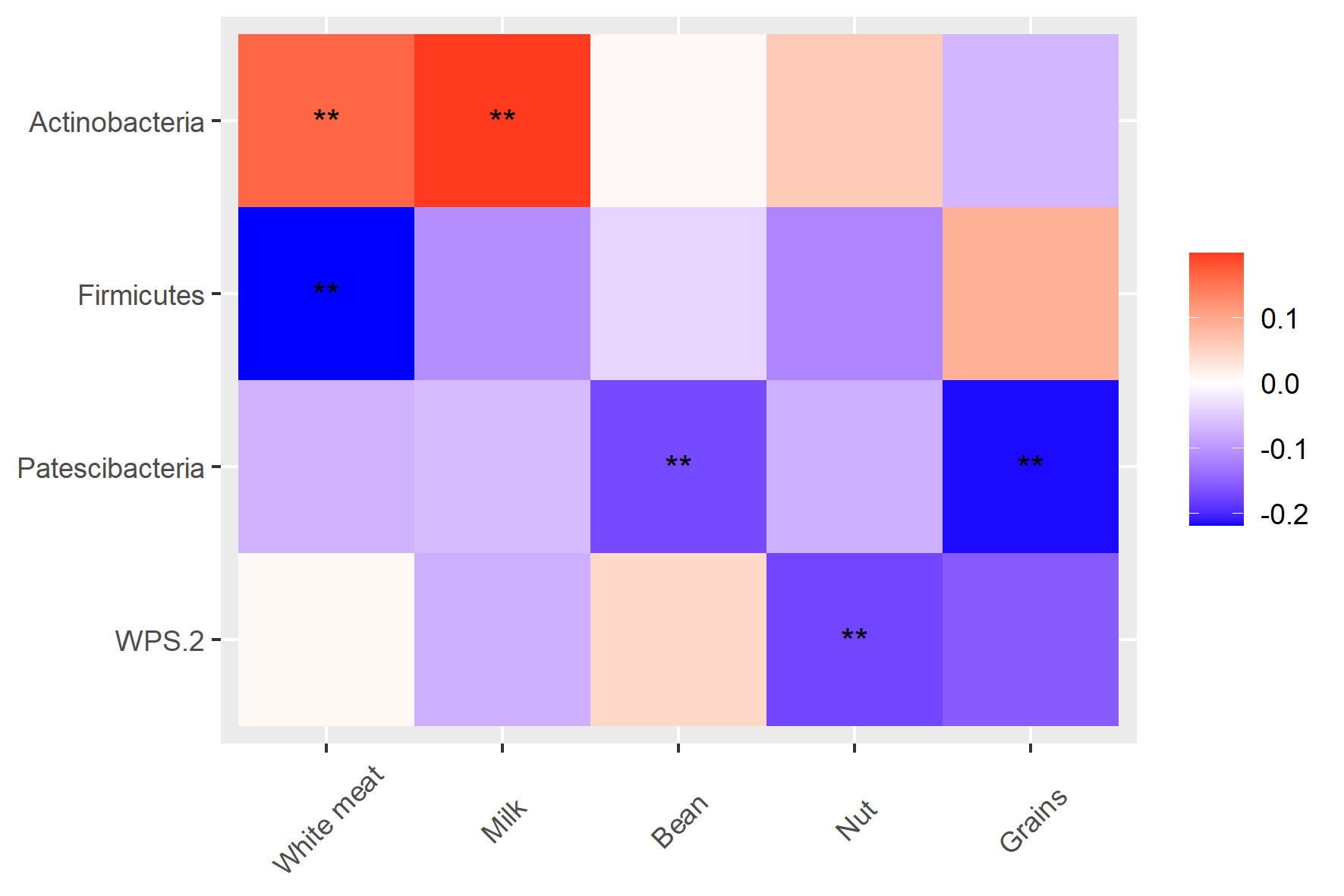

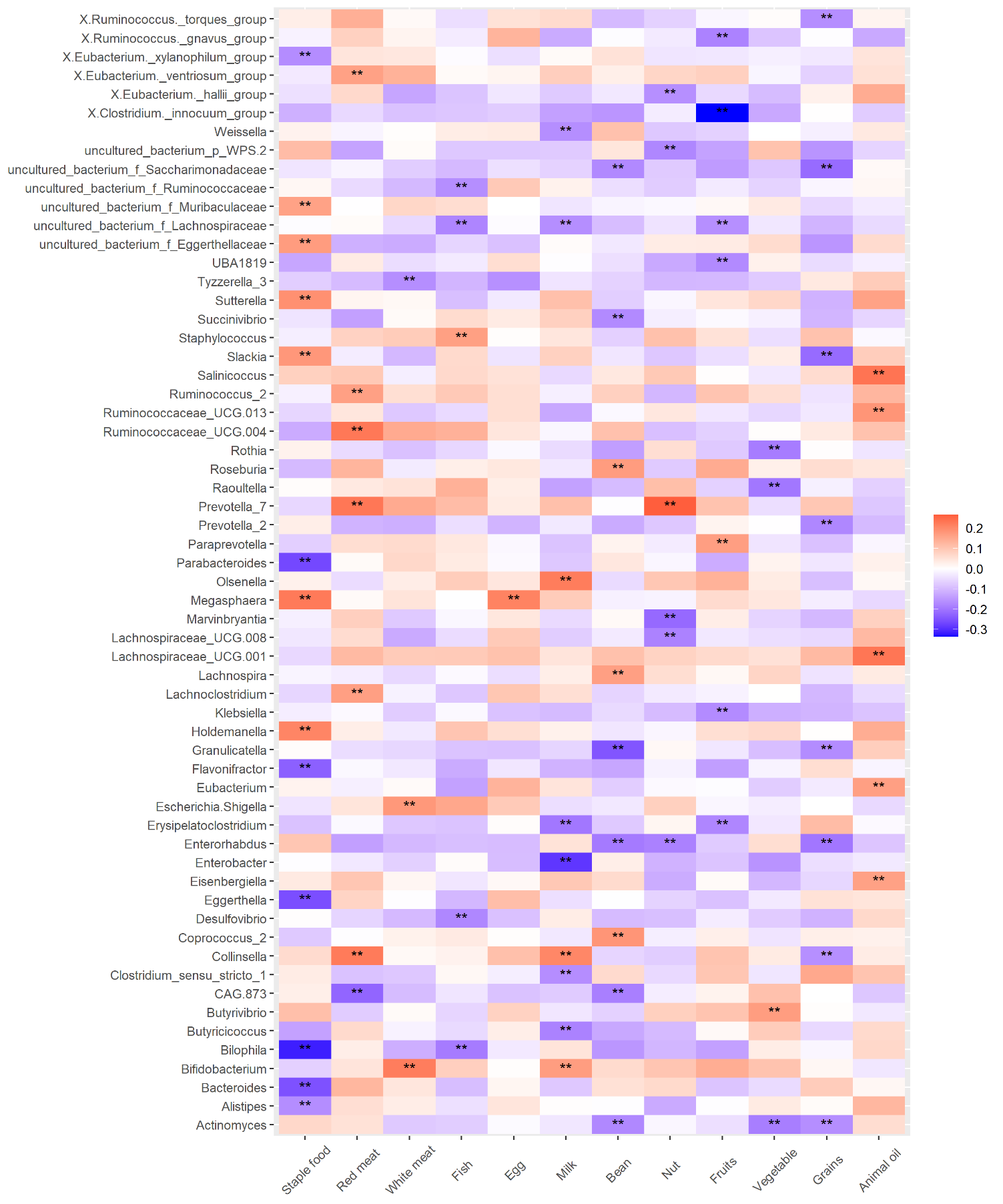

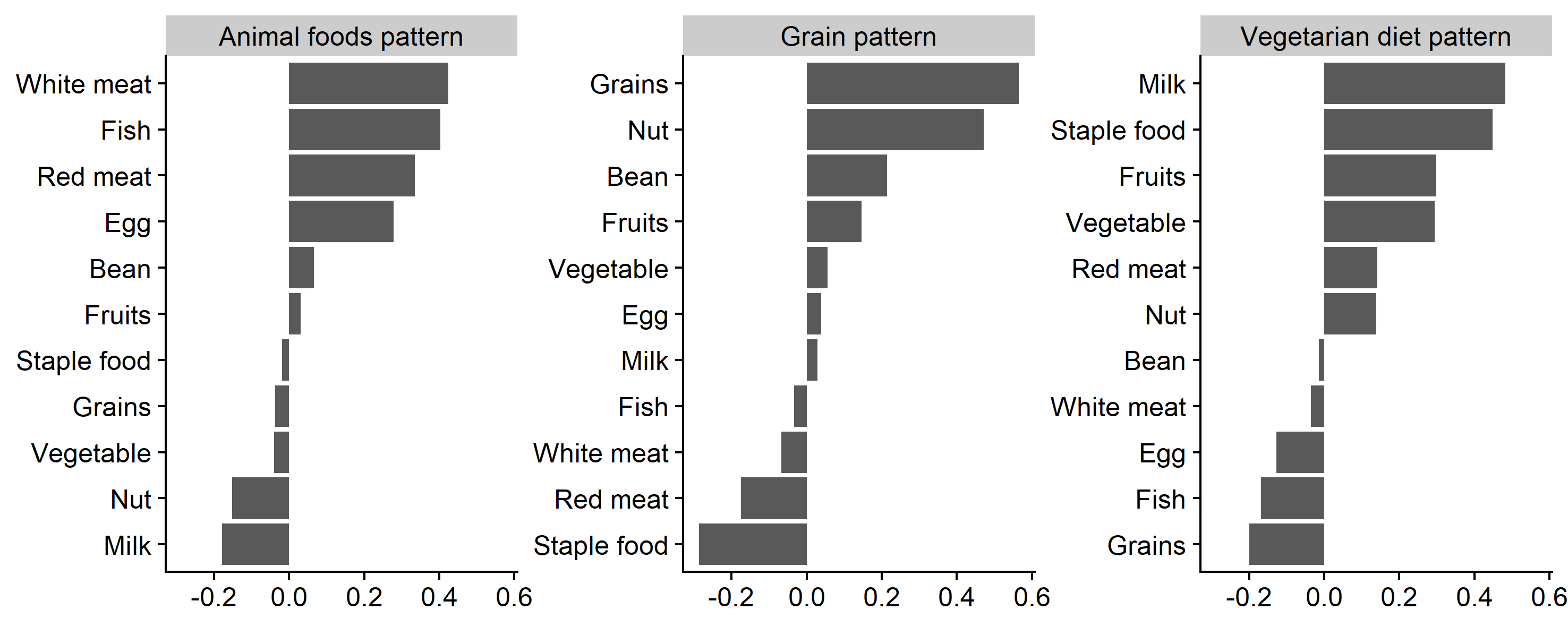


**Figure S1** Correlations between the intestinal microbiota at the genus levels and type 2 diabetes–related traits. Blocks in red indicate positive correlation, while blue blocks indicate negative correlations. The intensity of the colors represents the degree of association as measured by the Spearman correlation. ∗∗ indicates P < 0.05.

**Figure S2** Heat map of the Spearman correlation between dietary intake and intestinal microbiota. The intensity of the colors represents the degree of association as measured by the Spearman correlation. All significant correlations are marked with an asterisk (P <0.05).

**Figure S3** Correlations between the dietary intake and intestinal microbiota at the genus levels. Blocks in red indicate positive correlation, while blue blocks indicate negative correlations. The intensity of the colors represents the degree of association as measured by the Spearman correlation. ∗∗ indicates P < 0.05.

**Figure S4** Factor loadings for each food in the dietary pattern. The positive and negative values of the factor loadings indicate the direction of the correlation between food and dietary patterns, the greater the absolute value of the factor loadings, the greater the influence of food on dietary patterns.

## Supplementary Tables

| **Table S1 Comparison of intestinal microbiota composition between T2DM cases and controls** | | | | | |
| --- | --- | --- | --- | --- | --- |
| Taxonomic level | Name | T2DM | Control | *P* | *P*-adjusted |
| Class | Bacteroidia | 0.151 | 0.215 | 0.006 | 0.030 |
| Class | Erysipelotrichia | 0.017 | 0.009 | 0.002 | 0.030 |
| Class | Melainabacteria | 0.002 | 0.007 | 0.005 | 0.039 |
| Order | Bacteroidales | 0.151 | 0.215 | 0.006 | 0.042 |
| Order | Enterobacteriales | 0.076 | 0.054 | 0.007 | 0.036 |
| Order | Erysipelotrichales | 0.017 | 0.009 | 0.002 | 0.042 |
| Order | Pasteurellales | 0.001 | 0.002 | 0.008 | 0.035 |
| Family | Acidaminococcaceae | 0.006 | 0.009 | 0.001 | 0.013 |
| Family | Clostridiaceae_1 | 0.021 | 0.026 | 0.009 | 0.048 |
| Family | Enterobacteriaceae | 0.076 | 0.054 | 0.007 | 0.048 |
| Family | Enterococcaceae | 0.001 | 0.000 | 0.006 | 0.049 |
| Family | Erysipelotrichaceae | 0.017 | 0.009 | 0.002 | 0.028 |
| Family | uncultured_bacterium_o_Bacteroidales | 0.000 | 0.001 | 0.000 | 0.002 |
| Species | Enterobacter_ludwigii | 0.001 | 0.000 | 0.001 | 0.013 |
| Species | Streptococcus_mutans_UA159-FR | 0.000 | 0.000 | 0.005 | 0.047 |
| Species | bacterium_Te59R | 0.001 | 0.000 | 0.006 | 0.046 |
| Species | uncultured_bacterium_f_Peptostreptococcaceae | 0.004 | 0.003 | 0.001 | 0.018 |
| Species | uncultured_bacterium_g_Blautia | 0.061 | 0.044 | 0.001 | 0.017 |
| Species | uncultured_bacterium_g_Citrobacter | 0.002 | 0.000 | 0.003 | 0.033 |
| Species | uncultured_bacterium_g_Clostridium_sensu_stricto_1 | 0.018 | 0.026 | 0.000 | 0.013 |
| Species | uncultured_bacterium_g_Coprococcus_2 | 0.005 | 0.007 | 0.000 | 0.014 |
| Species | uncultured_bacterium_g_Fournierella | 0.000 | 0.000 | 0.001 | 0.017 |
| Species | uncultured_bacterium_g_Klebsiella | 0.027 | 0.005 | 0.001 | 0.015 |
| Species | uncultured_bacterium_g_Lachnospira | 0.001 | 0.002 | 0.000 | 0.002 |
| Species | uncultured_bacterium_g_Odoribacter | 0.000 | 0.000 | 0.006 | 0.049 |
| Species | uncultured_bacterium_g_Paraprevotella | 0.000 | 0.001 | 0.001 | 0.016 |
| Species | uncultured_bacterium_g_Phascolarctobacterium | 0.006 | 0.007 | 0.006 | 0.046 |
| Species | uncultured_bacterium_g_Roseburia | 0.011 | 0.022 | 0.001 | 0.013 |
| Species | uncultured_bacterium_g_Ruminococcaceae_UCG-010 | 0.001 | 0.001 | 0.005 | 0.049 |
| Species | uncultured_bacterium_g_Veillonella | 0.009 | 0.007 | 0.006 | 0.048 |
| Species | uncultured_bacterium_g_[Eubacterium]_xylanophilum_group | 0.000 | 0.000 | 0.000 | 0.016 |
| Species | uncultured_bacterium_o_Bacteroidales | 0.000 | 0.001 | 0.000 | 0.003 |
